# Role of Bcl-2 as a prognostic factor for survival in lung cancer: a systematic review of the literature with meta-analysis

**DOI:** 10.1038/sj.bjc.6601095

**Published:** 2003-07-01

**Authors:** B Martin, M Paesmans, T Berghmans, F Branle, L Ghisdal, C Mascaux, A-P Meert, E Steels, F Vallot, J-M Verdebout, J-J Lafitte, J-P Sculier

**Affiliations:** 1 Laboratoire d'Investigation Clinique H. J. Tagnon, Departement de Médecine, Institut Jules Bordet Brussels, Belgium; 2 Service d'Anatomie pathologique, Institut Jules Bordet Centre des Tumeurs de l'Université Libre de Bruxelles, Brussels, Belgium; 3 Service de Pneumologie, CHU Calmette, Lille, France

**Keywords:** Bcl-2, meta-analysis, survival, non-small cell lung cancer, small cell lung cancer, systematic review

## Abstract

The role of the anti-apoptotic protein Bcl-2 in lung cancer remains controversial. In order to clarify its impact on survival in small and non-small cell lung cancer (NSCLC), we performed a systematic review of the literature. Trials were selected for further analysis if they provided an independent assessment of Bcl-2 in lung cancer and reported analysis of survival data according to Bcl-2 status. To make it possible to aggregate survival results of the published studies, their methodology was assessed using a quality scale designed by the European Lung Cancer Working Party (including study design, laboratory methods and analysis). Of 28 studies, 11 identified Bcl-2 expression as a favourable prognostic factor and three linked it with poor prognosis; 14 trials were not significant. No differences in scoring measurement were detected between the studies, except that significantly higher scores were found in the trials with the largest sample sizes. Assessments of methodology and of laboratory technique were made independently of the conclusion of the trials. A total of 25 trials, comprising 3370 patients, provided sufficient information for the meta-analysis. The studies were categorised according to histology, disease stage and laboratory technique. The combined hazard ratio (HR) suggested that a positive Bcl-2 status has a favourable impact on survival: 0.70 (95% confidence interval 0.57–0.86) in seven studies on stages I–II NSCLC; 0.50 (0.39–0.65) in eight studies on surgically resected NSCLC; 0.91 (0.76–1.10) in six studies on any stage NSCLC; 0.57 (0.41–0.78) in five studies on squamous cell cancer; 0.75 (0.61–0.93) and 0.71 (0.61–0.83) respectively for five studies detecting Bcl-2 by immunohistochemistry with Ab clone 100 and for 13 studies assessing Bcl-2 with Ab clone 124; 0.92 (0.73–1.16) for four studies on small cell lung cancer; 1.26 (0.58–2.72) for three studies on neuroendocrine tumours. In NSCLC, Bcl-2 expression was associated with a better prognosis. The data on Bcl-2 expression in small cell lung cancer were insufficient to assess its prognostic value.

Lung cancer is the most common cause of cancer death in industrialised countries and its incidence is steadily increasing in women. Despite diagnostic and therapeutic improvements, the overall 5-year survival is still less than 15% (Laudanski *et al*, 1999).

A prognostic factor is a variable measured in individual patients that, alone or in combination with other factors, explains part of the population heterogeneity, and is at the time of diagnosis able to provide information on clinical outcome (Yip and Harper, 2000).

Some independent prognostic factors have been identified in order to predict survival and to help in the management of patients with lung cancer (Paesmans and Sculier, 1998). They include, for small cell lung cancer (SCLC), extent of disease and performance status (Paesmans *et al*, 2000), for resectable non-small cell lung cancer (NSCLC) performance status, TNM stage and age (Strauss, 1997); for advanced NSCLC, performance status, TNM staging, age, sex and weight loss (Buccheri and Ferrigno, 1994; Paesmans *et al*, 1995).

It has previously been reported that biological factors, angiogenesis (measurements of number of vessels per mm^2^), or factors reflecting proliferative state (number of cells in cycle) have a significant impact on survival in NSCLC (Kanters *et al*, 1995; O'Byrne *et al*, 2000). Unfortunately, these are relatively crude measures of the biological aggressiveness of the primary cancer because they involve several metabolic pathways.

Analysis and characterisation of proteins and genes involved in cancer development at the molecular level, could add to our knowledge of potential prognostic factors. These factors can be divided into categories according to their biological pathway: tumour suppressor genes, proto-oncogenes, markers of metastatic propensity, and proliferation markers (Strauss *et al*, 1995). Recent publications have attempted to correlate survival with factors related to angiogenesis (basic fibroblast growth factor, thrombospondin, vascular endothelial growth factor), to apoptosis (Bcl-2, p53), to control cell cycle (cyclins, MDM2, retinoblastoma gene), to growth (epithelial growth factor, erb-B2) and some other factors (serum lactate dehydrogenase (LDH), serum CYFRA21 level, white blood cell count and DNA aneuploidy content). The literature assessing their effects on survival (Strauss *et al*, 1995; Brambilla *et al*, 1996; Pujol, 1997; Kim *et al*, 1998; Kwiatkowski *et al*, 1998; D'Amico *et al*, 1999; Choma *et al*, 2001) remains controversial.

The Bcl-2 gene was originally discovered in a follicular B-cell lymphoma, where a chromosomal translocation t(14:18) moves the Bcl-2 gene into juxtaposition with transcriptional enhancer elements of the immunoglobulin heavy chain locus. (Tsujimoto and Croce, 1986; Aisemberg *et al*, 1988). In contrast, transregulatory mechanisms appear to be responsible for the high levels of Bcl-2 protein production that occur in many different solid tumours such as prostate cancer (Colombel *et al*, 2000), breast cancer (Silvestrini *et al*, 1994) and lung cancer (Pezzella *et al*, 1993; Fontanini *et al*, 1995). The Bcl-2 proto-oncogene is encoded by a 230 kb gene. Its product, a 26 kDa protein, is located in the inner mitochondrial membrane, and to a lesser extent in cell membranes (Jong *et al*, 1994). The major function of Bcl-2 appears to be to inhibit programmed cell death (apoptosis) and to prolong cell survival by arresting cells in the G_0_/G_1_ phase of the cell cycle. The ratio of death antagonists (Bcl-2, Bcl-X_L_, Bcl-W, Mcl-1, A1) to agonists (Bax, Bak, Bcl-X_s_, Bad, Bid) determines whether a cell will respond to an apoptotic signal. This death–life rheostat is mediated at least in part, by competitive dimerisations between selective pairs of antagonists and agonists (Kroemer, 1997). It is not clear from the data currently available as to which dimers are true regulators of apoptosis. Moreover, the possibility that at least some dimers form part of a regulatory higher-order, multiprotein complex cannot be excluded. The Bcl-2 protein is expressed in foetal tissues and basal cells of human epithelia, which suggests a role in normal growth regulation and differentiation (Hockenbery *et al*, 1990; Le Brun *et al*, 1993).

Although there are now a large number of studies of Bcl-2 expression, their value in predicting the survival of patients with lung cancer remains controversial. We have performed this systematic review of the literature to assess the prognostic value of Bcl-2 overexpression for the survival of lung cancer patients.

## MATERIALS AND METHODS

### Publication selection

To be eligible for inclusion in this systematic review, a study must have been published as a full paper in the English or French language literature and must meet the following criteria: deal with lung cancer only; analyse patients survival according to Bcl-2 status; measure Bcl-2 expression (protein, DNA or RNA) in the primary tumour (not in metastatic tissue or in tissue adjacent to the tumour) and/or antibodies against Bcl-2 in the serum.

An electronic search on Medline, using the keywords ‘lung neoplasms’ and ‘Bcl-2’, complemented by the personal bibliography of the authors, was used to select the articles. In addition, the bibliographies of studies already identified were used to complete trials identification. Studies published after December 1999 were not included.

Where the same author reported results obtained on the same patient population in several publications, only the most recent report, or the most complete one, was included in the analysis, in order to avoid overlap between cohorts.

### Methodological assessment

To assess methodology, 13 investigators (10 physicians, one pathologist, one biostatistician and one biologist) read each publication independently, and scored them according to the ELCWP scoring scale. The scoring system used in this literature review was used for a systematic review of the prognostic value of p53 on survival in lung cancer and has been previously reported (Steels *et al*, 2001).

Each item was assessed using an ordinal scale (possible values 2, 1, 0). The scores were compared and a consensus value for each item was reached in meetings attended by at least two thirds of the investigators. The participation of many readers was intended to facilitate correct interpretation of the articles.

The score evaluates a number of aspects of methodology, grouped into four main categories: scientific design, the description of laboratory methods used to identify the presence of Bcl-2 (protein, DNA/RNA or antibodies against Bcl-2), generalisability of results and the analysis of the study data. Each category had a maximum score of 10 points, giving a theoretical total maximum score of 40 points. The final scores were expressed as percentages, ranging from 0 to 100%, higher values reflecting better quality methodology. This allowed the value of ‘not applicable’ items to be discounted from the theoretical total of the relevant category.

### Statistical methods

A study was considered as significant if the *P*-value for the statistical test, comparing the survival distributions between the groups with and without Bcl-2 expression, was <0.05 in favour of this latter group. A study was classed as ‘positive’ when Bcl-2 expression was identified as an univariate indicator of good prognosis for survival. Other situations, were called ‘negative’, including the situation where a significant survival difference was found and the group of patients who were Bcl-2-positive fared worse.

The association between score measurements or between a score measurement treated as a continuous variable and another continuous variable was measured by the Spearman rank correlation coefficient. Its significance was assessed by testing a null hypothesis of equality to zero for this coefficient. The comparison between score measurement according to the value of a discrete variable was made by nonparametric Mann–Whitney (for dichotomic variables) or Kruskal–Wallis (for nominal variables with multiple classes) tests.

For the quantitative aggregation of the survival results, we measured the impact of Bcl-2 positivity on survival by hazard ratio (HR) between the survival distributions of the two Bcl-2 groups. For each trial, this HR was estimated by a method that depended on the results provided in the publication. The most accurate method was to retrieve the HR estimate and its variance from the reported results, or to calculate them directly using parameters given by the authors for the univariate analysis: the *O*−*E* statistic (difference between numbers of observed and expected events), the confidence interval for the HR, the log-rank statistic or its *P*-value. If these were not available, we looked for the total number of events, the number of patients at risk in each group and the log-rank statistic or its *P*-value, allowing calculation of an approximation of the HR estimate. Finally, if the only useful data were in the form of graphical representations of the survival distributions, we extracted from them survival rates at specified times in order to reconstruct the HR estimate and its variance, with the assumption that during the study follow-up the number patients counted was constant (Parmar *et al*, 1998). If authors reported survival of three or more groups (e.g., using several cutoff values for percentage of protein present in the cytoplasm, or regarding the exons of DNA separately), we pooled the results in order to make a comparison between two groups feasible.

Global survival of the entire patient population was analysed, when available. If not, the results of subgroups were treated separately. If survival was reported separately for particular subgroups, these results were treated in the meta-analysis of the corresponding subgroups. The same patients were never considered more than once in each analysis. The individual HR estimates were combined into an overall HR using the method published by Peto (Yusuf *et al*, 1985). By convention, an HR<1 implied a better survival for the group with positive Bcl-2. This impact of Bcl-2 on survival was considered as statistically significant if the 95% confidence interval (CI) for the overall HR did not overlap 1.

For the subgroups where heterogeneity was detected by *χ*^2^ tests for heterogeneity, a calculation of the overall effect using a random-effects model was also included.

The studies eligible for the systematic review were called ‘eligible’ and those providing data for meta-analysis ‘evaluable’.

## RESULTS

### Studies selection and characteristics

A total of 29 trials, published between 1993 and 1999, were selected (Pezzella *et al*, 1993; Fontanini *et al*, 1995, 1996; Walker *et al*, 1995; Brambilla *et al*, 1996; Kaiser *et al*, 1996; Ohsaki *et al*, 1996; O'Neill *et al*, 1996; Rao *et al*, 1996; Takayama *et al*, 1996; Anton *et al*, 1997; Apolinario *et al*, 1997; Higashiyama *et al*, 1997; Ishida *et al*, 1997; Koukourakis *et al*, 1997; Pastorino *et al*, 1997; Greatens *et al*, 1998; Kim *et al*, 1998; Kwiatkowski *et al*, 1998; Chen *et al*, 1999; D'Amico *et al*, 1999; Dingemans *et al*, 1999; Dosaka-Akita *et al*, 1999; Eerola *et al*, 1999; Ghosh *et al*, 1999; Huang *et al*, 1999; Laudanski *et al*, 1999; Maitra *et al*, 1999; Santinelli *et al*, 1999). They all report on the prognostic value for survival of Bcl-2 status in lung cancer patients, assessing Bcl-2 protein expression in the primary tumour. One study was excluded because an identical patient cohort was used in another selected publication (references excluded/included: (Fontanini *et al*, 1996)/(Fontanini *et al*, 1995)).

The main features of the 28 studies eligible for the systematic review are shown in Table 1

**Table 1:** Main characteristics and results of the eligible studies

			**NSCLC**									
	**All studies**		**Any stage**		**Locoregional (I–II)**		**Surgical treatment (I–III)**		**SCLC**		**Neuroendocrine tumours**	
	**Total**	***S***	**Total**	***S***	**Total**	***S***	**Total**	***S***	**Total**	***S***	**Total**	***S***
Number of studies	28 (25)	11 (11)	7 (6)	2 (2)	6 (5)	2 (2)	8 (7)	6 (6)	4 (4)	0	3 (3)	1 (1)

NSCLC=non-small cell lung cancer; SCLC=small cell lung cancer; *S*=number of studies identifying Bcl-2 positivity as a statistically significant good prognostic factor; ( )=number of studies evaluable for meta-analysis.

. A total of 21 trials looked at NSCLC, while SCLC and neuroendocrine tumours were studied in four and three trials respectively. Non-small cell lung cancer trials included either all histological subtypes (*n*=17), or adenocarcinoma (*n*=2) or squamous cell cancer (*n*=2). Data related to patients treated by surgery (stages I–IIIB) comprised eight of the 21 NSCLC trials. Six of the 21 NSCLC studies were performed in locoregional disease (stages I–II), while seven were dealt with any stage (stages I–IV).

Immunohistochemistry techniques (IHC) were used in all the trials to detect the expression of Bcl-2 protein. Various antibodies were used to assess Bcl-2 expression. The two clones most used were clones 100 and 124, in 25% (seven out of 28) and 71% (20 out of 28) of the studies respectively.

Three of the 28 trials eligible for the systematic review reported insufficient data for the HR to be evaluable for the quantitative aggregation. The reasons for not including studies in the meta-analysis were as follows: no survival curve shown (*n*=1) (Kwiatkowski *et al*, 1998); no *P*-value, HR or CI reported (*n*=1) (Greatens *et al*, 1998); no proportion of Bcl-2 positive (*n*=1) (Greatens *et al*, 1998; Dosaka-Akita *et al*, 1999).

### Studies results report

As shown in Table 1, 11 of the 28 studies (39.3%) identified Bcl-2 expression as a good prognostic factor for survival (all evaluable for meta-analysis), 14 (50%) concluded that Bcl-2 was not a prognostic factor for survival (11 evaluable) and three (10.7%) linked Bcl-2 expression with poor prognosis (three evaluable,).

Of the 21 published NSCLC trials, 11 (57.1%) were positive. All of these studies were evaluable for meta-analysis. None of the four studies dealing with SCLC reported significant results. One of the three concerning neuroendocrine tumours was significant.

Evaluability status for the meta-analysis was associated with trial positivity: the rate of positive results was 44% for evaluable trials (11 out of 25) compared to 0% (zero out of three) for nonevaluable ones (*P*=0.26).

### Quality assessment

Overall, the global quality assessment score, expressed as a percentage, ranged between 32.9 and 79.1%, with a median of 54.6% (Table 2A

**Table 2:** Methodological assessment by ELCWP score, according to trials characteristics: (A) all trials and (B) evaluable trials for meta-analysis

		**Global score (%)**	**Design (/10)**	**Laboratory methodology (/10)**	**Generalisability (/10)**	**Results analysis (/10)**
(A) *All trials*						
						
Total (*n*=28)	All studies	54.6	4.0	6.1	6.7	5.0
	Patient number *P*-value	**0.0015**	**0.03**	**0.03**	**0.03**	**0.008**
	Evaluable for the MA (*n*=25)	54.6	4.0	6.4	6.7	5.0
	Not evaluable for the MA (*n*=3)	54.2	5.0	7.8	6.7	5.0
	*P*-value	0.53	0.28	0.17	0.91	0.50
	Positive (*n*=11)	51.5	4.0	5.7	6.6	5.0
	Negative (*n*=17)	54.6	5.0	7.14	6.6	5.0
	*P*-value	0.27	0.07	0.44	0.48	0.94
	IHC Ab clone 100 (*n*=5)	59.4	4.0	5.0	6.7	7.5
	IHC Ab clone 124 (*n*=21)	52.1	4.0	6.1	6.7	5.0
	*P*-value	0.25	0.81	0.54	0.54	0.08
						
(B) *Evaluable trials for meta-analysis*						
Evaluable for the MA (*n*=25)	All studies	54.6	4.0	6.4	6.7	5.0
	Patient number *P*-value	**0.004**	0.09	**0.03**	**0.045**	**0.01**
	Positive (*n*=11)	51.5	4.0	5.7	6.7	5.0
	Negative (*n*=14)	57.0	5.0	6.8	7.1	5.6
	*P*-value	0.35	**0.01**	0.64	0.53	0.80
	IHC Ab clone 100 (*n*=5)	59.4	4.0	5.0	6.7	7.5
	IHC Ab clone 124 (*n*=18)	50.3	4.0	5.7	6.7	5.0
	*P*-value	0.10	0.07	0.37	0.07	1.0

Score distributions are summarised by median values. Positive=studies identifying Bcl-2 positivity as significant good prognostic factor for survival; negative=studies reporting nonsignificant results, or associating Bcl-2 positivity with poor survival; MA=meta-analysis; IHC=immunohistochemistry. The values in bold were significant.

where only the median values are shown). The design subscore had the lowest values. The most poorly described items (<30% of the maximum) were the *a priori* estimate of sample size required to conduct the study, the outcome definition, the double-blinding evaluation of the biological marker, the reproducibility control test between the experimenters and the initial disease work-up description.

A weak but significant correlation between the global score and the number of patients included in the study was observed (Spearman's correlation coefficient *r*=0.56, *P*=0.0015).

No statistically significant difference was found between the 25 evaluable and the three nonevaluable studies either for the global score (median 54.6% in comparison to 54.2%, *P*=0.53 by the Mann–Whitney test), or for the four subgroups scores.

There was also no statistically significant difference between the global scores of 11 positive trials and the 17 negative trials (median 51.5% in comparison to 54.6%, *P*=0.27 by Mann–Whitney test), nor for their four subscores.

The score difference between the studies classified according to the types of monoclonal antibody used was not significant. The overall median score was respectively 59.4 and 52.1% when clone 100 or clone 124 antibodies were used (*P*=0.25 by Mann–Whitney test).

Table 2B describes the scores for the 25 trials classified as evaluable for meta-analysis. Their overall quality score ranged between 32.9 and 79.1%, with a median of 53.9%. There was a significant correlation between the global score and the number of patients included in the study (Spearman's correlation coefficient *r*=0.55, *P*=0.004). The scores of the four subgroups matched those of the 28 studies, with the design subscore again being the worse reported. The most poorly described items (<30% of maximal score) were the *a priori* estimate of sample size required to conduct the study, the outcome definition, the double-blinding evaluation, the reproducibility control test between the experimenters, the initial disease work-up description and the number of unassessable samples, with the reason for their exclusion. There was no significant difference between positive and negative trials in their global score with a median of 51.5 and 57.0% respectively for the positive and the negative studies (*P*=0.35).

The type of monoclonal antibody did not affect the overall quality assessment, which had a median global score of 59.4% for clone 100 and of 50.3% for clone 124 (*P*=0.10).

### Meta-analysis

The absence of any significant qualitative difference between positive and negative trials allowed us to perform a quantitative aggregation of the survival data. However, only subgroup analysis could be performed due to the heterogeneity of the trials: the trials authors had reported on patients with different histological subtypes (NSCLC, SCLC or neuroendocrine tumours); stages (localised, locoregional or extensive); or treatments. The subgroups were defined according to histology, extent of the disease, technique used to detect Bcl-2 (IHC with the two most frequently used monoclonal antibodies clone 124 and 100) and the threshold used to determine Bcl-2 positivity.

The hazard ratios were retrieved by one of the three methods reported in the Materials and methods section. Only four studies reported the data necessary to estimate the HR directly. In eight trials, the HR was approximated using the total number of events and the log-rank statistic or its *P*-value. For the 13 remaining studies, the HR was extrapolated from the graphical representations of the estimated survival distributions.

In all, 28 eligible trials analysed overall survival in relation to Bcl-2 expression in 3829 patients. Three trials were excluded and thus the analysis was restricted to 3370 patients (88%).

Overall, Bcl-2 protein was expressed in 39% of the lung tumours studied: 71% in SCLC, 55% in neuroendocrine tumours and 35% in NSCLC. In the NSCLC group, 32% of the squamous cell cancer and 61% of the adenocarcinoma expressed Bcl-2. Bcl-2 expression was found in 23, 37 and 50% respectively for the subgroups of patients with stage I–II, surgically treated stage I–III, and any stage disease.

The NSCLC subgroup included 18 trials comprising 2909 patients. The aggregated survival data showed a good survival prognosis where there was Bcl-2 positivity (HR=0.72; 95% CI 0.64–0.82).

Stages I and II NSCLC subgroup included eight trials comprising 1311 patients. The aggregation produced a statistically significant HR of 0.70 (95% CI 0.57–0.86) (Table 3

**Table 3:** Meta-analysis of the subgroup including studies of stages I and II NSCLC with their characteristics

**Group of NSCLC: stages I–II (*n*=8)**							
**Study**	**Method**	**Threshold**	**QS (%)**	**N Pts**	**Bcl-2+ (%)**	**HR**	**95% CI**
Apolinario *et al* (1997)	IHC-clone 100	NM	59	73	51	0.40	0.16–0.98
Chen *et al* (1999)	IHC-clone 124	NM	33	40	43	0.18	0.04–0.88
D'Amico *et al* (1999)	IHC-clone 120	>50	72	408	23	0.88	0.60–1.28
Higashiyama *et al* (1997)	IHC-clone 124	>10	52	38	39	0.30	0.06–1.62
Koukourakis *et al* (1997)	IHC-clone 100	NM	58	107	19	0.32	0.13–0.86
Ohsaki *et al* (1996)	IHC-clone 124	>20	50	45	29	0.45	0.13–1.56
Pastorino *et al* (1997)	IHC-clone 120	>10	76	485	17	0.89	0.65–1.21
Pezzella *et al* (1993)	IHC-clone 100	NM	65	115	22	0.54	0.29–1.03
							
Overall (fixed-effects model)				1311	23	0.70	0.57–0.86
Overall (random-effects model)						0.59	0.42–0.83
*χ*^2^ statistic for heterogeneity=12.80, 7 df, *P*=0.08							

IHC-clone 100=immunohistochemistry with monoclonal antibody 100; IHC-clone 124=immunohistochemistry with monoclonal antibody 124; QS=Median quality score; N pts=number of patients; Bcl-2+=presence of Bcl-2; df=degree of freedom; HR=hazard ratio; CI=confidence interval; NM=not clearly mentioned.

). The result of the test for heterogeneity was not significant (*P*=0.08), but it was not possible to go further in categorising the trials, and to treat separately papers reporting on stage I patients. The use of a random-effects model did not change the conclusion, with a combined HR of 0.59 (95% CI 0.42–0.83). The surgically treated NSCLC (NSCLC completely removed by surgery for stages I–IIIB), with seven out of eight trials evaluable, showed, a significant HR of 0.50 (95% CI 0.39–0.65) (Table 4

**Table 4:** Meta-analysis of the subgroup including studies performed in NSCLC treated by surgery, with their characteristics

**Group of NSCLC: surgical stages (*n*=7)**							
**Study**	**Method**	**Threshold**	**QS (%)**	**N Pts**	**Bcl-2+ (%)**	**HR**	**95% CI**
Fontanini *et al* (1995, 1996)	IHC-clone 124	>1	45	89	66	0.28	0.14–0.55
Ghosh *et al* (1999)	IHC-clone 124	>50	41	134	31	0.60	0.40–0.90
Higashiyama *et al* (1997)	IHC-clone 124	>10	52	174	21	0.47	0.20–1.14
Huang *et al* (1999)	IHC-clone 124	>50	70	203	39	0.46	0.26–0.82
Ishida *et al* (1997)	IHC-clone 124	>10	64	114	38	0.23	0.06–0.86
Kim *et al* (1998)	IHC-clone 124	NM	79	NM	NM	2.50	0.90–7.1
Laudanski *et al* (1999)	IHC-clone 124	NM	68	84	46	0.41	0.21–0.79
							
Overall (fixed-effects model)				798	37	0.50	0.39–0.65
Overall (random-effects model)						0.50	0.33–0.77
*χ*^2^ statistic for heterogeneity=14.98, 6 df, *P*=0.02							

The meaning of the symbols is described in Table 3.

). Once again, the introduction of a random effect did not change the interpretation of the HR (HR 0.50; 95% CI 0.33–0.77). The subgroup of studies including any stage NSCLC had an HR of 0.91 (95% CI 0.76–1.10) (Table 5

**Table 5:** Meta-analysis of the subgroup including studies performed in any stage of NSCLC, with their characteristics

**Group of NSCLC: all stages (*n*=6)**							
**Study**	**Method**	**Threshold**	**QS (%)**	**N Pts**	**Bcl-2+ (%)**	**HR**	**95% CI**
Anton *et al* (1997)	IHC-clone 124	>10	49	427	47	0.87	0.70–1.07
Kim *et al* (1998)	IHC-clone 124	NM	79	238	72	1.80	1.1–2.9
O'Neill *et al* (1996)	IHC-clone 124	>1	55	54	35	1.34	0.53–3.44
Ohsaki *et al* (1996)	IHC-clone 124	>20	50	96	17	0.46	0.22–0.98
Rao *et al* (1996)	IHC-clone 124	NM	45	41	61	0.63	0.22–1.84
Walker *et al* (1995)	IHC-clone 124	>50	46	27	44	0.18	0.03–0.97
							
Overall (fixed-effects model)				883	50	0.91	0.76–1.10
Overall (random-effects model)						0.85	0.53–1.37
*χ*^2^ statistic for heterogeneity=15.34, 5 df, *P*=0.009							

The meaning of the symbols is described in Table 3.

).

Five trials for squamous cell cancer were assessable (Table 6

**Table 6:** Meta-analysis of the studies performed in squamous cell cancer, with their characteristics

**Group of squamous cell cancer (*n*=5)**							
**Study**	**Method**	**Threshold**	**QS (%)**	**N Pts**	**Bcl-2+ (%)**	**HR**	**95% CI**
Chen *et al* (1999)	IHC-clone 124	NM	33	40	42	0.18	0.03–0.88
Ghosh *et al* (1999)	IHC-clone 124	>50	41	134	31	0.6	0.40–0.89
Higashiyama *et al* (1997)	IHC-clone 124	>10	52	67	31	0.32	0.08–1.25
O'Neill *et al* (1996)	IHC-clone 124	>1	55	54	35	1.34	0.52–3.44
Pezzella *et al* (1993)	IHC-clone 100	NM	65	75	27	0.42	0.20–0.91
							
Overall (fixed-effects model)				370	32	0.57	0.41–0.78
Overall (random-effects model)						0.54	0.33–0.89
*χ*^2^ statistic for heterogeneity=6.67, 4 df; *P*=0.15							

The meaning of the symbols is described in Table 3.

). Results were significantly in favour of Bcl-2 positivity with an HR of 0.57 (95% CI 0.41–0.78).

**Figure 1:**
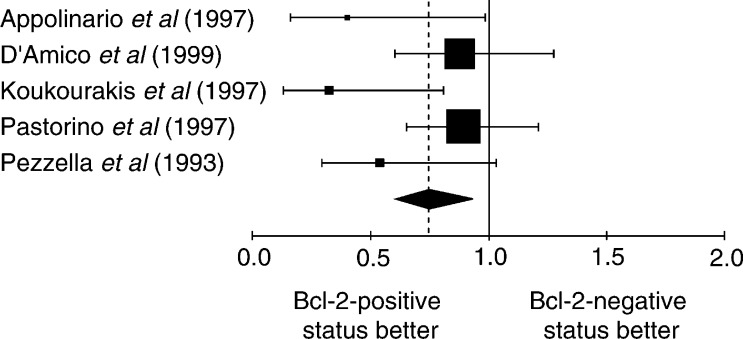
Hazard ratio (HR) and 95% CI of mortality in studies evaluating Bcl-2 status by IHC with Ab 100. *χ*^2^ statistic for heterogeneity=8.14, 4 df, *P*=0.09. NB: HR<1 implies a survival benefit for the group with positive Bcl-2. The square size is proportional to the number of patients included in the study. The centre of the lozenge gives the combined HR of the meta-analysis and its extremities the 95% CI.

The NSCLC group was meta-analysed according two further criteria: the method used to detect Bcl-2 overexpression and the Bcl-2 positivity threshold. The aggregated results are shown in Figures 1

, 2

**Figure 2:**
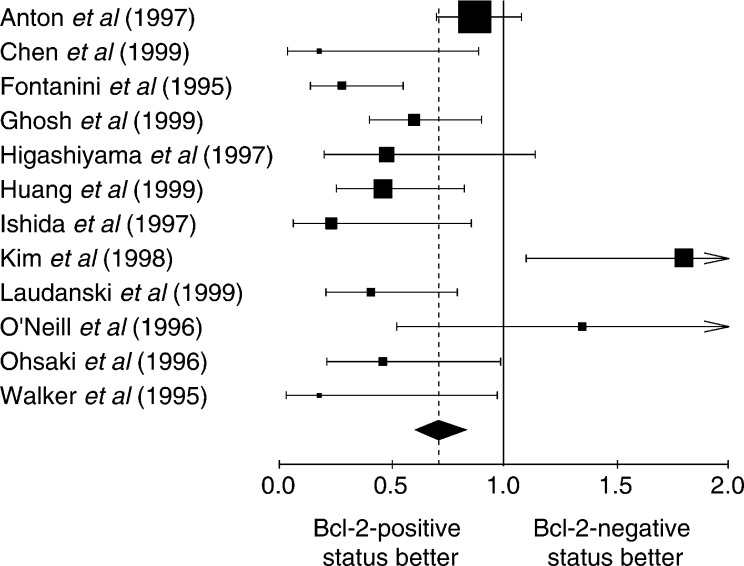
Hazard ratio and 95% CI of mortality in studies evaluating Bcl-2 status by IHC with Ab 124. The meaning of the symbols is described in Figure 1.

, 3

**Figure 3:**
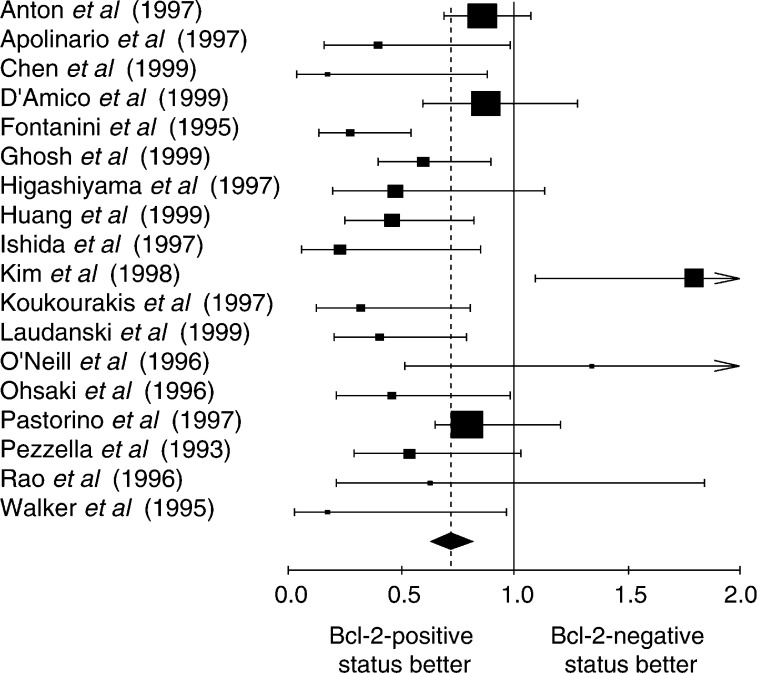
Hazard ratio and 95% CI of mortality in studies incorporating NSCLC whatever the threshold of positivity chosen by the authors. The meaning of the symbols is described in Figure 1.

, 4

**Figure 4:**
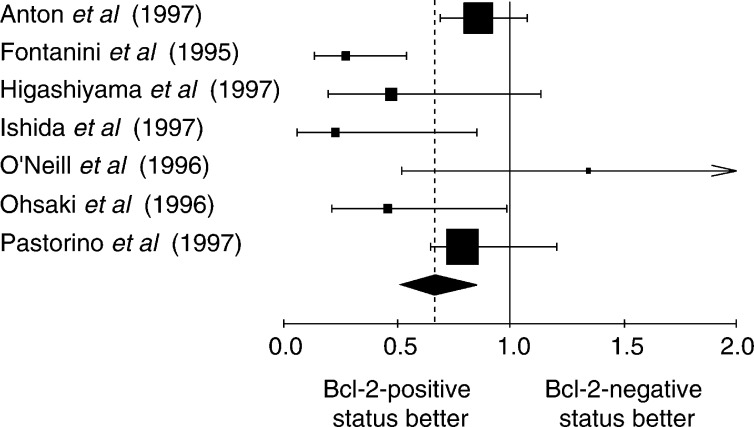
Results of the meta-analysis for the subgroup of studies where a tumour was considered as expressing Bcl-2 if 1–20% of the cells were positive for Bcl-2. The meaning of the symbols is described in Figure 1.

, 5

**Figure 5:**
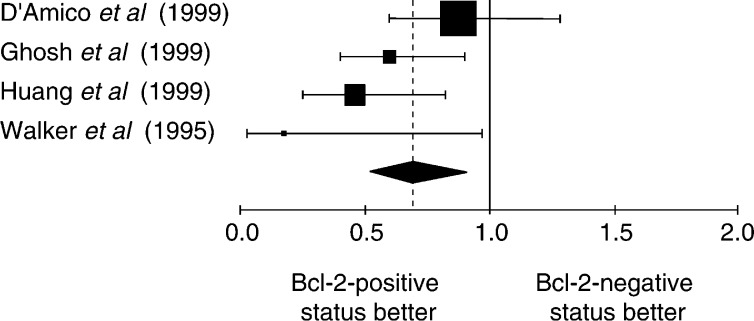
Results of the meta-analysis for the subgroup of studies where a tumour was considered as expressing Bcl-2 if 21–50% of the cells were positive for Bcl-2. The meaning of the symbols is described in Figure 1.

and 6

**Figure 6:**
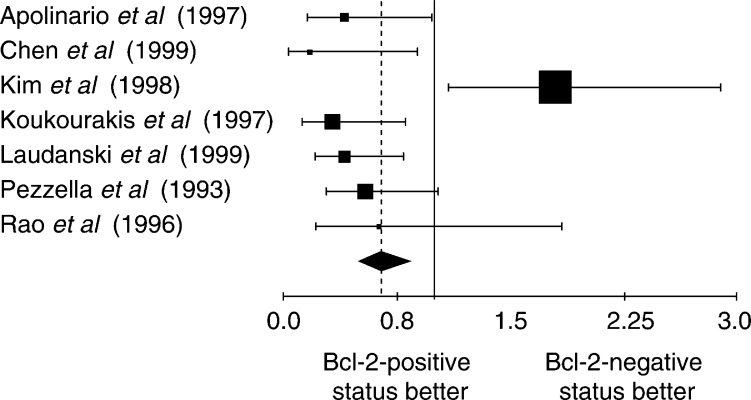
Results of the meta-analysis for the subgroup of studies where a tumour was considered as expressing Bcl-2 if the percentage of the cells was not clearly mentioned positive for Bcl-2. The meaning of the symbols is described in Figure 1.

. The individual data from these studies have been reported in Tables 3, 4, 5 and 6. Firstly, the monoclonal antibodies clones used according to HR for the studies assessing Bcl-2 with antibody clone 100 and clone 124 were respectively 0.75 (95% CI 0.61–0.93) and 0.71 (95% CI 0.61–0.83). Secondly, the studies were divided into four groups according to the definition of the threshold for Bcl-2 positivity: global group, threshold from 1 to 20%, threshold up to 50% and threshold not clearly described. HR, calculated by a fixed-effect model, were respectively 0.73 (95% CI 0.64–0.82), 0.77 (95% CI 0.66–0.91), 0.65 (95% CI 0.51–0.83) and 0.62 (95% CI 0.52–0.91).

In the SCLC subgroup, four studies (all reported as negative) comprised together 317 patients. The aggregation produced an HR of 0.92 (95% CI 0.73–1.16) (Table 7

**Table 7:** Meta-analysis of the studies performed in small cell lung cancer, with their characteristics

**Group of SCLC (*n*=4)**							
**Study**	**Method**	**Threshold**	**QS (%)**	**N Pts**	**Bcl-2+ (%)**	**HR**	**95% CI**
Dingemans *et al* (1999)	IHC-clone 100	>10	67	91	78	1.55	0.93–2.58
Kaiser *et al* (1996)	IHC	>50	63	146	75	0.76	0.55–1.04
Maitra *et al* (1999)	IHC-clone 100	>10	46	42	57	0.68	0.36–1.27
Takayama *et al* (1996)	IHC-clone 124	>10	55	38	55	1.31	0.64–2.70
							
Overall (fixed-effects model)				317	71	0.92	0.73–1.16
Overall (random-effects model)						0.99	0.66–1.48
*χ*^2^ statistic for heterogeneity=7.35, 3 df, *P*=0.06							

The meaning of the symbols is described in Table 3. IHC=Immunohistochemistry with other antibodies than clone 100 and 124.

).

For the three studies dealing with neuroendocrine tumours (86 patients), the aggregation produced an HR of 1.26 (95% CI 0.58–2.72) (Table 8

**Table 8:** Meta-analysis of the studies performed in neuroendocrine tumoral lung cancer, with their characteristics

**Group of neuroendocrine tumoral lung cancer (*n*=3)**							
**Study**	**Method**	**Threshold**	**QS (%)**	**N Pts**	**Bcl-2+ (%)**	**HR**	**95% CI**
Brambilla *et al* (1996)	IHC-clone 124	>50	41	43	56	11.48	1.34–98.01
Eerola *et al* (1999)	IHC-clone 124	NM	45	20	18	0.44	0.13–1.43
Santinelli *et al* (1999)	IHC-clone 124	NM	49	23	61	1.81	0.57–5.77
							
Overall (fixed-effects model)				86	55	1.26	0.58–2.72
Overall (random-effects model)						1.71	0.35–8.41
*χ*^2^ statistic for heterogeneity=6.79, 2 df, *P*=0.03							

The meaning of the symbols is described in Table 3.

).

## DISCUSSION

Our systematic review of the literature shows that overexpression of the Bcl-2 protein is a good prognostic factor for survival in patients with NSCLC. The analysis reveals similar features in different subgroups of localised NSCLC and clarifies the message of individual studies that are somewhat inconsistent.

The decision to perform the meta-analysis was based on a prior methodological assessment of the publications. We have used a methodology similar to previous systematic reviews reported by our group on the treatment of lung cancer (Luce *et al*, 1998; Sculier *et al*, 1998; Meert *et al*, 1999; Mascaux *et al*, 2000) after an adaptation to biological prognostic factors such as p53 (Steels *et al*, 2001). By comparing the scores of the studies where Bcl-2 was a significant prognostic factor and those where it was not, we could identify differences, suggesting biases induced by trial methodology. Nevertheless, our approach does not eliminate all potential biases.

First, we have to consider publication bias. Our review took into account only fully published studies. We did not look for unpublished trials and abstracts because the methodology we used required data that are usually only available in full publications. Meta-analysis based on data on individuals is considered by some authors as the gold standard (Stewart and Parmar, 1993). Systematic reviews of the literature and meta-analyses of individual patient data should not be confused. The first approach is based only on fully published studies and provides an exhaustive and critical analysis of the topic with an adequate methodology based on the criteria of Mulrow (1987) and with data aggregation (meta-analysis) when possible. The second approach is, in fact, a new study taking in all trials performed on the topic, whether published or not. It requires that the investigators update individual data. In the latter case, publications are used mainly for identification purposes. In prophylactic cranial irradiation, our meta-analysis (Meert *et al*, 2001), based on the published data, yielded the same results for patients in complete remission as Aupérin *et al* (1999) showed in their individual data meta-analysis. This supports the validity of our approach. Our review deals with studies of prognostic factors and, as they are most often retrospective, it is much more difficult to identify unpublished data than it is with clinical trial data. Furthermore, we were not able to include all the papers identified in the meta-analysis due to under-reported results, which occurred more often in papers where an effect of Bcl-2 on survival was not shown.

The comparison of the score of the two groups (positive and negative trials) showed no statistically significant difference, allowing a meaningful data aggregation. The three studies excluded from the meta-analysis due to a lack of reported data were all negative. There is, thus, a potential bias in favour of positive trials. It should, however, be stressed that results were significantly better reported in the positive studies than in the negative ones. Indeed, studies with no statistically significant results are less often published or, if they are published, it is with more concise reports of results, meaning that they are more often unassessable. Moreover, there is a language bias. We have restricted our review to articles published in English and French, because all our readers did not know other languages such as Japanese or German. This bias could favour the positive studies that are more often published in English, while the negative ones are more often reported in native languages (Egger *et al*, 1997).

Another potential source of bias is related to the method for extrapolating the HR. If they were not reported by the authors, HR were calculated from the data available in the article and, if that was not possible, they were extrapolated from the survival curves, which involves making assumptions. Moreover, there is no consensus over the choice of time intervals for reading survival rates on the curves. Finally, we would emphasise that a global meta-analysis did not appear meaningful because of the heterogeneity of the patient populations. The patient population of the studies available is very heterogeneous, often they were restricted to patients with a specific histological subtype or a selected tumour stage. For this reason, we did not perform a global analysis and instead focused our analysis on more homogeneous subgroups of patients by aggregating data from studies conducted in similar patient populations or on similar tumours. When using a random-effects models, we came to the same conclusions as we did with fixed-effects models. However, such models do not identify the source of the heterogeneity, itself an important clinical point. It was not possible, on the basis of published data, to adjust our results in a multivariate analysis.

Our results are based on an aggregation of data obtained by univariate survival analysis in retrospective trials. The results need to be confirmed by an adequately designed prospective study and the exact value at which Bcl-2 should be considered ‘overexpressed’ determined by an appropriate multivariate analysis taking into account the classical well-defined prognostic factors for lung cancer. A meta-analysis based on the individual data of the patients included in studies (Stewart and Parmar, 1993) would help to define by multivariate methods the prognostic role of Bcl-2, but it would require the collection of a huge amount of retrospective data, with the potential problem of dealing with a lot of missing data. But such a study could never have the equivalent value of a well-designed prospective study (Cappelleri *et al*, 1996).

Another possible source of confusion is the use of same cohort of patients for different publications (Fontanini *et al*, 1996). If the same patients are included twice or more in a meta-analysis, it may give a higher weighting to these studies. In the systematic review, we have excluded the studies for which it was possible to identify with certainty that similar patients cohorts had been used in different publications (Fontanini *et al*, 1995). On the other hand, when the data in the publication did not allow us to decide if the same cohort of patients was being investigated (Pezzella *et al*, 1993; Koukourakis *et al*, 1997), we have assumed that the authors have been sufficiently honest not to re-report the results from the same cohort of patients without making this clear in the paper.

Finally, for practical purposes, and because of their small number, we have included in the negative group the three trials that showed that of the presence of Bcl-2 had a significant negative effect on survival.

The techniques used to identify overexpression of Bcl-2 status can also be a potential source of bias. The IHC used to reveal the Bcl-2 protein is not always performed with the same antibody. Sometimes the protocol was performed without prior reaction of epitope unmasking on fixed issue (Cattoretti *et al*, 1993). To try to exclude technical biases, we performed subgroup analysis according to the most frequently used methods: IHC with antibody clone 100 and clone 124 (Figure 1 and Figure 2). In both cases, the results were consistent with a favourable survival in the case of Bcl-2 overexpression, making it improbable that the techniques were a source of bias. Moreover, the cutoff in the number of positive cells defining a tumour with Bcl-2 overexpression is often arbitrary and varies according to the investigators, from a few percent to 50%. The use of different cutoff points for IHC is of critical importance, as was shown by Lee *et al* (1995). Some investigators selected the cutoff point based on the minimum *P*-value approach, which can lead to seriously biased conclusions (Altman *et al*, 1994). If a chosen cutoff is often arbitrary, selection according to the median value of expression levels provides a more standardised approach to prognostic factors, although it may lead to some loss of information (Altman *et al*, 1994). An optimal threshold still needs to be defined for Bcl-2.

It should be noted that the four eligible studies reporting on SCLC and two of the three studies concerning neuroendocrine tumours were negative. In fact, it is very difficult to draw a definite conclusion because of the small number of patients included in these trials. Consequently, further studies are necessary to determine the value of Bcl-2 as a prognostic factor for survival in SCLC and in neuroendocrine tumours.

In our systematic review with meta-analysis, patients with Bcl-2-positive tumours had significantly better survival than those with Bcl-2-negative tumours. The mechanism underlying the effect of Bcl-2 oncoprotein expression on tumour progression and prognosis remains essentially uncertain. Originally, the Bcl-2 gene product was implicated in oncogenesis because of its ability to prolong cell survival through the inhibition of apoptosis (Adams and Cory, 1998; Antonsson and Martinou, 2000). The process of apoptosis involves many proteins such as the antiapoptotic proteins (Bcl-2, Bcl-X, Bfl-1) and the proapoptotic proteins (Bax, Bak, Bad) (Kroemer, 1997). These proteins can interact in order to regulate cellular apoptosis by balancing pro- and antiapoptotic mechanisms. Thus, the study of only one apoptotic protein produces an incomplete appraisal of apoptosis and it would be interesting to conduct a survival analysis of a combination of these proteins. Moreover, the distribution of Bcl-2 protein observed in normal tissues and embryonic tissues indicates that it has a function in morphogenesis linked to cell proliferation via escape from cell death (Le Brun *et al*, 1993; Adams and Cory, 1998; Antonsson and Martinou, 2000). In NSCLC, Fontanini *et al* (1995) stated that Bcl-2 oncoprotein expression status was not correlated with proliferative potential indicators including PCNA and Ki-67. On the other hand, considering how rarely extrathoracic metastasis in NSCLC express Bcl-2, it could be proposed that this oncoprotein plays an inhibitory role in the haematogenous metastatic process through tumour progression. The question of whether Bcl-2 oncoprotein biologically participates in the haematogenous metastatic process and reduces the incidence of distant metastasis has still to be elucidated.

In conclusion, our systematic review of the lung cancer literature suggests that overexpression of Bcl-2, in patients with NSCLC has good prognostic value for survival, whatever the biological test used. This observation is potentially important. Identification of independent prognostic factors allows us to define high-risk patients for whom specific therapy may be designed or to introduce stratification in randomised trials. In lung cancer, the prognostic factors currently used are clinical variables such as performance status or disease extent. The results of our meta-analysis, which suggest a relation between Bcl-2 and survival, should encourage properly designed prospective studies, with an appropriate statistical methodology including multivariate analysis, in order to demonstrate the usefulness of molecular biological markers like Bcl-2, assessed by IHC.
